# Synaptic transistor with multiple biological functions based on metal-organic frameworks combined with the LIF model of a spiking neural network to recognize temporal information

**DOI:** 10.1038/s41378-023-00566-4

**Published:** 2023-07-21

**Authors:** Qinan Wang, Chun Zhao, Yi Sun, Rongxuan Xu, Chenran Li, Chengbo Wang, Wen Liu, Jiangmin Gu, Yingli Shi, Li Yang, Xin Tu, Hao Gao, Zhen Wen

**Affiliations:** 1 School of Advanced Technology, Xi’an Jiaotong-Liverpool University, Suzhou, 215123 P.R. China; 2 Department of Electrical Engineering and Electronics, University of Liverpool, Liverpool, L69 3GJ UK; 3 School of Science, Xi’an Jiaotong-Liverpool University, Suzhou, 215123 P.R. China; 4 Department of Electrical Engineering, Eindhoven University of Technology, Den Dolech 2, 5612 AZ Eindhoven, The Netherlands; 5 Institute of Functional Nano and Soft Materials (FUNSOM), Joint International Research Laboratory of Carbon-Based Functional Materials and Devices, Soochow University, Suzhou, 215123 P.R. China

**Keywords:** Electronic devices, Electronic properties and materials

## Abstract

Spiking neural networks (SNNs) have immense potential due to their utilization of synaptic plasticity and ability to take advantage of temporal correlation and low power consumption. The leaky integration and firing (LIF) model and spike-timing-dependent plasticity (STDP) are the fundamental components of SNNs. Here, a neural device is first demonstrated by zeolitic imidazolate frameworks (ZIFs) as an essential part of the synaptic transistor to simulate SNNs. Significantly, three kinds of typical functions between neurons, the memory function achieved through the hippocampus, synaptic weight regulation and membrane potential triggered by ion migration, are effectively described through short-term memory/long-term memory (STM/LTM), long-term depression/long-term potentiation (LTD/LTP) and LIF, respectively. Furthermore, the update rule of iteration weight in the backpropagation based on the time interval between presynaptic and postsynaptic pulses is extracted and fitted from the STDP. In addition, the postsynaptic currents of the channel directly connect to the very large scale integration (VLSI) implementation of the LIF mode that can convert high-frequency information into spare pulses based on the threshold of membrane potential. The leaky integrator block, firing/detector block and frequency adaptation block instantaneously release the accumulated voltage to form pulses. Finally, we recode the steady-state visual evoked potentials (SSVEPs) belonging to the electroencephalogram (EEG) with filter characteristics of LIF. SNNs deeply fused by synaptic transistors are designed to recognize the 40 different frequencies of EEG and improve accuracy to 95.1%. This work represents an advanced contribution to brain-like chips and promotes the systematization and diversification of artificial intelligence.

## Introduction

Compared with traditional chips, nonvolatile neural devices have competitive advantages which includes low energy consumption and high-speed parallel operation. Computing in memory (CIM) has the same protocols and standards for storage and memory, which is the top research for neuromorphic computing^1^. In recent years, resistive random access memories (RRAMs) as memristors have been integrated with microprocessors and peripheral circuits to realize the artificial intelligence (AI) functionalities of neural networks^2,3^. The NeuRRAM-a chip is an advanced RRAM-based CIM chip that offers comparable inference accuracy to software models with four-bit weights for various AI tasks. It also boasts energy efficiency that is twice as good as previous state-of-the-art RRAM-CIM chips across different computational bit-precisions. Additionally, the NeuRRAM-a chip allows for flexible reconfiguration of CIM cores to accommodate diverse model architectures^4,5^. From the perspective of energy consumption, three-terminal neural devices have more potential to approach the power of the human brain (25 W) in large-scale computing^6^. However, due to the limitations and deficiencies of array fabrication technology for three-terminal neural devices, synaptic transistors as cross-bar weights combined with functional circuits are rarely explored to completely simulate the neural network^7^. Many studies have focused on the synaptic plasticity of a single device and the nonvolatile regulation mechanism^3,8^. Consequently, the bionic performance of the synaptic transistor is utilized to expand the fusion circuit and match the high-performance network, which has a great contribution to accelerating the improvement of the brain-like computing system^9,10^.

The essence of brain-like computing is learning from the information processing method or structure of biological neural systems and then developing matching computer theory, chip architecture, and application models and algorithms^11^. Brain-like computing is considered to be a significant research avenue in the post-Moore era, which has the potential to break through a technological bottleneck in future intelligent computing^12^. At present, spiking neural networks, which closely replicate biological nervous systems, are a promising technology due to their low-overhead online learning and energy-efficient information encoding, stemming from their intrinsic local training principles. Thus, the comprehensive deepening innovation of spiking neural networks (SNNs) must be explored in all related fields, including model algorithms, software, chips, and data^13^. Several multiterminal synaptic devices, including floating-gate synaptic transistors (STs), ferroelectric-gate STs, electrolyte-gate STs, and optoelectronic STs, have been developed for producing synaptic plasticity. This plasticity is classified based on factors such as the retention time and the number of pulses. These devices effectively provide the ability to manipulate synaptic strength^14,15^. The working principles of the above STs, including thermal emission or quantum tunneling, promote electrons into the floating gate. The interaction between the carriers in the channel and the polarization of the ferroelectric insulator is known as the Coulomb interaction, electrostatic modulation and electrochemical doping and interfacial charge trapping through photogenerated electron pairs. Moreover, the functional layer, comprising a variety of materials (metal oxides, organic materials, two-dimensional, quantum dots and perovskite), can enhance or expand the synaptic properties of a system with regard to energy consumption, computing speed, and compatibility^16^. However, to date, AI applications of metal-organic frameworks (MOFs) in nonvolatile neural devices have rarely been reported. MOFs are a type of crystalline porous material that are created by combining polytopic organic ligands with metal centers. These MOFs possess several advantageous characteristics, such as highly ordered pores, a substantial surface area, and a modifiable structure, which conveniently makes designing controlled and multifunctional biological spiking neural devices uncomplicated. Furthermore, by deeply introducing the core unit of SNNs, SNNs commonly adopt leaky integration and firing (LIF) neurons as the fundamental building blocks for constructing neural networks^17,18^. The LIF neuron model is a well-known type of neuron that offers a combination of the user-friendliness and simplicity of the integrate-and-fire (IF) model, along with the capability to simulate various physiological properties of biological neurons, similar to the Hodgkin-Huxley (H-H) neuron model^19^. For synaptic devices, the LIF model is computationally efficient due to its simplicity, making it suitable for large-scale simulations. Moreover, the LIF model is biologically plausible and can simulate a wide range of physiological properties of biological neurons, such as action potential generation, synaptic integration, and adaptation^20^. For the AI application, the LIF model is compatible with a range of learning rules (long-term potentiation/long-term depression (LTP/LTD) and spiking-timing-dependent plasticity (STDP)) and can be used to train SNNs for various tasks, such as classification, pattern recognition, and control. In particular, studies on constructing LIF neuron circuits and composing the forward propagation process of SNNs with output signals from synaptic weight cross-bars have been rarely reported^21^. Therefore, to overcome the barrier from the extraction of single device characteristics to the building of an integral neural network system, more resources are needed to form the complete neuromorphic system^22^. In terms of operation speed, the appropriate data type is conducive to improving the working efficiency of the neural network^23^. In addition, the advantage of SNNs is to process complex temporal information that has obvious differences in the frequency domain^6^. The steady-state visually evoked potential (SSVEP) is a neural reaction that occurs in response to visual stimuli^24^. When the eyes receive periodic flashes of light, the brain generates a stable electrical signal that oscillates at the same frequency as the stimulus. This response can be recorded via electroencephalography (EEG) and is typically observed as a periodic waveform at a specific frequency. SSVEP has been widely used in the development of brain-computer interfaces (BCIs); these systems enable individuals to control external devices by monitoring their brain activity^25^. For instance, in an SSVEP-based BCI system, users can select different commands or controllers by fixating on visual stimuli that flash at distinct frequencies on a computer screen. The system identifies the choice of the user by analyzing their EEG and executes the corresponding operation. SSVEP-based BCIs have diverse applications in fields such as virtual reality, game control, and medical diagnosis^24,26^.

In this work, we have proposed a new type of spiking neural network that utilizes a ZIF-67 synaptic transistor, LIF neuron circuits, and SSVEPs to achieve efficient and accurate neural computations. Forward propagation in our network relies on time sequence coding, accumulation of postsynaptic current, and the membrane potential threshold voltage of LIF neurons. Backpropagation in the proposed SNNs involves determining the iteration update rules and integrating the STDP curve to adjust the synaptic weights between neurons. The functional diversity of the prepared artificial neurons can be clearly observed through the results of STM/LTM, paired-pulse facilitation (PPF), STDP, and LTP/LTD. More importantly, an LIF circuit capable of producing a matching array output has been simulated, allowing the SNNs to efficiently convert high-frequency information into sparse signals using the four blocks. Ultimately, the task of recognizing EEG signals was achieved using the modified SNN, with the final recognition rate stabilizing at 95.2%.

## Results and discussion

### Physical and electrical characterization

**Fig. 1:**
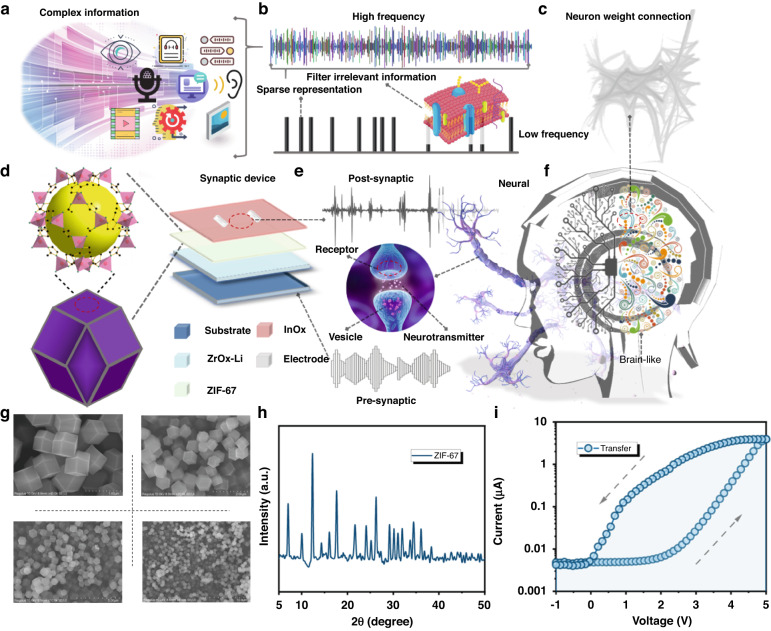
Human brain processing complex information, the relationship between biological and electrical synapses, and the basic characterization of synaptic transistors. **a** Schematic diagram depicting the complex information encompassed by the analog signals. **b** The human brain processes high-frequency information through sparse representation to obtain filtered low-frequency information. **c** Low-energy operating modes in the human brain are facilitated by neurons connected through different weighted nodes. **d** Structure diagram of the synaptic transistor and basic crystal of ZIF-67. **e** A biological chemical synapse is illustrated schematically, comprising a presynaptic terminal, a receptor, and a postsynaptic terminal. **f** Various functional areas in the human brain are composed of billions of neural networks. **g** SEM images of ZIF-67. **h** XRD patterns of ZIF-67. **i** Channel current dependence of the gate voltage analyzed at a V_post_ of 0.5 V

Neuromorphic computing research has been pursued to approach the computational power of the human brain (Fig. 1a). The human brain receives a vast amount of information every day and can quickly process and identify the features of things^27^. This information can be a vast information parameter obtained through human sensory organs such as sound, mechanical, visual, and touch. Low-energy processing is because the scale of information received by the brain is filtered. High-frequency information is transformed into high-precision sparse low-frequency information after passing through neurons (Fig. 1b). These pieces of information can strongly stimulate the synaptic function of the brain, enabling it to quickly activate connections between neurons for the next input of the same information. Neurons that trigger the same event are connected by multiple synapses with different weights^4,28^. The weight values and the number of synapses connecting each node to the next node are different. Thus, a neural network composed of approximately 80 billion neurons can handle heavy parallel data (Fig. 1c). To promote the systematization of three-terminal neuromorphic devices, we constructed a modifiable synaptic device with MOFs as the main functional layer (Fig. 1d). Compared with two-dimensional materials, organic materials and metal oxide materials as the partial structure of synaptic transistors, MOF materials have the advantages of high porosity, excellent light-stimulated synaptic plasticity properties, good stability in the atmosphere, and synthetic tunability. The device is composed of the following layers, from top to bottom: source/drain, InO_x_, ZIF-67, ZrO_x_, substrate, and gate. The ZIF-67 layer, acting as the trapping layer, can capture and release carriers to change the conductance in the channel when a positive/negative voltage is applied to the gate. To provide a vivid explanation of the relationship between biological synapses and electronic synapses, we liken the process of converting chemical signals into electrical signals from the presynaptic terminal to the postsynaptic terminal (neurotransmitters released by synaptic vesicles are accepted by receptors on the membrane) to applying a pulse to the gate of the device and receiving a corresponding pulse between the source/drain (excitatory postsynaptic current (EPSC)) (Fig. 1e). The synaptic structure and neurons in the human brain are the main basis for brain-like devices (Fig. 1f). To clearly display the MOF structure of ZIF-67, we analyzed scanning electron microscopy (SEM) images at four different resolutions (Fig. 1g). These results provide valuable insights into the design of neuromorphic computing devices and the potential for energy-efficient processing. The SEM images of the as-prepared ZIF-67 sample reveal a uniform size distribution with a well-defined cubic morphology, demonstrating excellent dispersion and a solid interior. These observations provide valuable insights into the structural characteristics of the MOF material and its potential for use in neural applications, including neuromorphic computing devices. The uniform size and cubic morphology of the ZIF-67 particles suggest that they could offer excellent stability and reproducibility in device fabrication processes. Additionally, the well-defined morphology and solid interior of the particles suggest that they could provide a high surface area-to-volume ratio, potentially enhancing their performance in various applications. Overall, the SEM images provide important information about the structural properties of ZIF-67 and its potential for use in a wide range of AI and neuromorphic applications. The crystalline structure of the ZIF-67 sample was analyzed using X-ray diffraction (XRD), which revealed that the main characteristic peaks of the bare ZIF-67 matched well with those reported in the literature (Fig. 1h). These results indicated the successful synthesis of the MOF material on the ZrO_x_ substrate^5,9^. The XRD analysis was further supported by the SEM characterization, which demonstrated that the ZIF-67 particles exhibited a uniform size and cubic morphology with good dispersion and a solid interior. The successful synthesis of ZIF-67 on the ZrO_x_ substrate was confirmed by both SEM and XRD characterizations. Figure 1i illustrates the transfer characteristics of synaptic devices, where the channel current increases as the applied voltage increases from −1 V to 5 V (V_DS_ = 2 V), exhibiting typical n-type transfer behavior^29^. The application of a higher positive gate voltage results in the migration of more cations from the electrolyte into the porous MOF channel, leading to n-type doping of the MOF channel and an increase in its conductivity^16^. The porous nature of MOFs allows for easy penetration of cations into the channel under low gate voltage, thereby enabling the ZIF-67 synaptic device to operate as a low-voltage transistor with low power consumption.

### Electrical measurement of synaptic plasticity

**Fig. 2:**
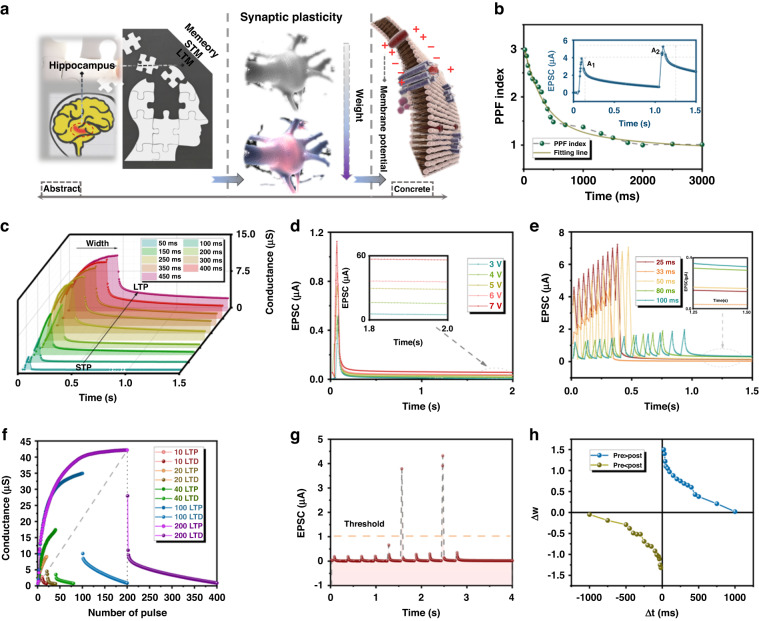
Three biological functions and corresponding synaptic characteristics. **a** The device simulates three typical functions of synapses: forming memory, synaptic plasticity, and stimulating membrane potential. **b** The PPF index is a measure of synaptic facilitation defined as the ratio of the amplitudes of the first (A_1_) and second (A_2_) EPSCs plotted against the pulse interval (${\Delta\mathrm{t}}$). **c** EPSC behaviors activated and modified by electric pulses with 9 different widths (50 ms, 100 ms, 150 ms, 200 ms, 250 ms, 300 ms, 350 ms, 400 ms, and 450 ms) at V_DS_ = 0.5 V. **d** EPSC triggered by 5 single electric pulses with different amplitudes (3 V, 4 V, 5 V, 6 V, and 7 V). **e** Low-pass filtering characteristics are shown by 10 continuous pulses of different frequencies (10 Hz, 12.5 Hz, 20 Hz, 30 Hz, and 40 Hz) applied to the presynaptic terminal. **f** The LTP/LTD characteristics demonstrate the controllable range and level of conductance. **g** Threshold effect of the synaptic device as biological neurons. **h** Trend of the STDP curve as the weight update rule transforms the temporal information

To demonstrate the three typical neuronal functions of the proposed synaptic transistor, standard electrical tests are performed for validation (Fig. 2a). First, the short-term memory/long-term memory (STM/LTM) of the human brain primarily originates from the hippocampus, which is the foundation of memory and the basis of all intelligent life^30^. The hippocampus also promotes frequent association of events and forgetting unimportant information. Second, synaptic weight and plasticity refer to the strength or amplitude of the connection between two nodes, which in biology corresponds to the amount of influence that one neuron has on another node through its discharge^7,12^. Third, biological neurons only transmit stimuli to other neurons they are connected to when they receive external stimuli that exceed a certain threshold, thereby facilitating information exchange through membrane potential. The occurrence of paired pulse facilitation (PPF) is associated with the release of neurotransmitters by presynaptic neurons and is typically believed to be regulated by calcium. By examining the paired pulse facilitation (PPF) index, we investigated the synaptic plasticity features of the ionotronic synaptic transistor, which play a crucial role in recognizing visual signal information in biological neural systems. Paired input spikes with various time intervals (∆t) are used to trigger the PPF index (A_2_/A_1_), and the resulting secondary EPSC peak (A_2_) is compared to the first peak (A_1_) to determine the presence of facilitation behavior. In Fig. 2b, the generated A_2_ is 301% higher than A_1_ when the time interval is 20 ms with 1.5 V. The PPF index reflects the extent of synaptic connection enhancement between neurons and can be simulated using the double-exponential function.

1 $${\rm{PPF}}={{\rm{C}}}_{0}+{{\rm{C}}}_{1}\,{{\rm{e}}}^{\left(-\frac{\Delta{\rm{t}}}{{{\uptau}}1}\right)}+{{\rm{C}}}_{2}\,{{\rm{e}}}^{\left(-\frac{\Delta{\rm{t}}}{{{\uptau}}2}\right)}$$

The initial constants of the rapid and slow phases C_0_, C_1_, and C_2_ are 1, 15%, and 33%. The relaxation times are$\,{\tau }_{1}\,$(15 ms) and ${\tau }_{2}$ (20 ms). In addition, the PPF curve under other different voltages (1 V and 2 V) shows controllable biological characteristics under different external environments (Fig. S1). To accurately assess the impact of a single pulse on synaptic plasticity, we analyzed the effects of electric pulses with different widths and amplitudes on EPSC excitation. We measure the dynamic current behaviors responsive to gate voltage pulses with different widths (50 ms to 450 ms) and the same amplitude of 1.5 V (Fig. 2c). To demonstrate the conductance retention characteristics of the synaptic device, Fig. S2 shows the fluctuation of the currents within 30 min and 90 min. After reaching a peak value, the EPSC returns to its original current state, indicating that the peak values are proportional to the amplitude of the voltage pulses^15^. Nevertheless, it has been observed that pulses with a width greater than 50 ms do not fully return to their original state, indicating significant nonvolatile properties. This behavior is similar to that seen in biological excitatory synapses. Additionally, low power consumption is crucial for developing an energy-efficient neuromorphic chip. By multiplying the peak value of the EPSC, the drain voltage, and the pulse duration, it is estimated that the power of a single spike generated by the gate voltage of −1 V is 1.8 nJ^31^. To further discuss the effect of temporal properties on synaptic plasticity, we demonstrate the EPSCs responsive to gate voltage pulses with different amplitudes (3 to 7 V) and the same duration of 50 ms (Fig. 2d). Because the mechanism of the proposed synaptic transistor is ion migration, the pulse width applied at the gate can enhance the synaptic plasticity of the device more than the pulse amplitude. To further verify the effect of ion migration on synaptic plasticity, Fig. S3 shows the EPSC when the doping concentration is 5%. Ion doping into the ZrO_x_ layer plays an important role in lowering the range of the pulse width. Curiously, the ZIF-67 synaptic transistor has the characteristic of filtering high-frequency information, which is similar to the LIF neuron (Fig. 2e). Furthermore, this high-frequency filtering phenomenon is closer to the function of the human brain. The human brain can process massive amounts of information at very low power every day. This is attributed to the fact that high-frequency information does not cause neurons to generate high-frequency pulses. In short, having the characteristics of high-frequency filtering better matches the algorithm strategy in the SNN. Detailed investigations have been carried out on the characteristics of LTP/D, which are essential features for synaptic operation in neuromorphic computing. The various conductance states of synaptic devices are demonstrated in Fig. 2f, where a sequence of excitatory spikes (V_DS_ = +1 V, t_d_ = 50 ms, ∆t = 100 ms) and inhibitory spikes (V_DS_ = −1 V, t_d_ = 50 ms, ∆t = 100 ms) were applied. The peak current increased gradually from 8.9 μA to 42.5 μA as the quantity of pulses rose from 20 to 100 and recovered to the initial level under application of the excitatory and inhibitory spikes, respectively^17^. The LTP/LTD curves show the conductance margins (G_max_/G_min_) between the maximum and minimum conductance values as a function of the number of spike pulses. Different ratios (3.809, 14.481, 22.905, 52.620, and 53.36) indicate the resolution between synaptic weight updates and the corresponding learning step size (Fig. S4). Compared with other neural devices, the larger G_max_/G_min_ ratio of this work can make the update range of synaptic weights wider. In neuromorphic computing, the iterative update rule between neurons is constrained by weights represented by the conductance of the synaptic device^20^. To further verify the membrane potential threshold of synaptic transistors, we applied continuous pulses (V_DS_ = +0.5 V, t_d_ = 50 ms, ∆t = 200 ms) that avoid the potentiation of conductance (Fig. 2g). As observed from the red line, the transient accumulation of ion migration in MOFs can result in the release of an instantaneous pulse exceeding the threshold (1 μA). The measured alteration in synaptic weight following each neuron spiking event is depicted in Fig. 2h using the synaptic device structure. An increase (decrease) in synaptic weight occurs when the preneuron spikes before (after) the postneuron^32^. Moreover, the synaptic weight change with respect to the spike timing difference (∆t) can be accurately described by exponential decay functions, confirming that the STDP properties are similar to those observed in biological synaptic systems. It is apparent that upon approaching ∆t = 0 from ∆t = 0.1 s, the synaptic weight is potentiated. For ∆t < 0, the synaptic weight is depressed. This behavior is known as the asymmetric Hebbian learning rule. Furthermore, we measured the extreme conditions that trigger STDP to ensure that the neural device can be efficiently and continuously updated (Fig. S5).

### LIF as SNN neurons

**Fig. 3:**
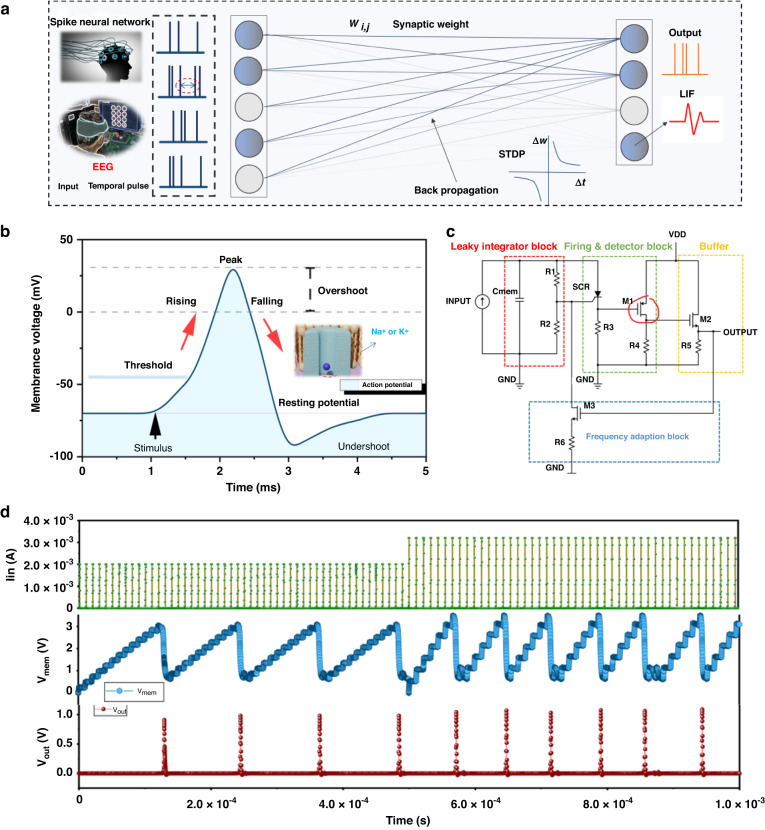
SNN framework and LIF model combined with EPSC. **a** Block diagram of feedforward and back propagation for the spiking neural network based on the LIF neuron model and STDP weight update rule. **b** Fluctuation area of membrane voltage in biological neurons. **c** The leaky integrator block, fire and detector block, buffer block, and frequency adaptation block are combined with the output of ZIT-67 synaptic devices to form the complete LIF system. **d** Operation of the VLSI circuit with input pulses of the synaptic device (t_on_ = 100 ns, t_rise_ = t_fall_ = 1 ns, period = 2 μs, I_in_ = 2.0 mA and 3.3 mA)

The desire to replicate the remarkable energy efficiency of biological systems has been a significant driving force behind the advancement of spiking neural networks (SNNs) (Fig. 3a). One main theory for the superior energy efficiency of SNNs is their significantly higher information capacity when compared to other neural network models, such as the multilayer perceptron, which is based on firing rates^8^. In contrast to SNNs, training firing-rate networks often involves the use of backpropagation algorithms, which can be challenging to implement efficiently due to the centralized method for computing weight updates and the need for large amounts of high-precision memory^32^. In SNNs, the LIF neuron model is commonly used to simulate the behavior of neurons. The LIF model can simulate how neurons receive and process signals from other neurons and fire a spike when a certain threshold is reached. Additionally, the leaky integration mechanism in the LIF model can mimic the gradual decrease in membrane potential in biological neurons over time. STDP is a key mechanism in SNNs that describes how the strength of synapses between neurons changes over time based on the timing of their spike activities. Fig. S6 shows the LIF model of standard SNNs with random input spikes (50% firing probability). Synaptic weights are updated through the STDP mechanism to simulate learning and adaptation between neurons. Together, the LIF neuron model and STDP mechanism play crucial roles in SNNs, enabling the simulation of neuronal activity and plasticity. Fig. S7 demonstrates the 3-layer improved spiking neural network based on the STDP of the synaptic transistor. The LIF neuron model simulates the changes in membrane potential (V_m_) of a neuron over time (Fig. 3b). The V_m_ changes are determined by the flow of ions through various channels in the neuron’s membrane^12,19^. Initially, the neuron is at rest with a resting membrane potential. When a presynaptic neuron sends a signal to the postsynaptic neuron, it causes a small increase in V_m_, known as the postsynaptic potential (PSP). If enough PSPs are received, V_m_ reaches a threshold voltage, at which point an action potential or spike is generated and sent down the axon of the neuron. After a spike is generated, the neuron enters a refractory period where it cannot generate another spike, known as the absolute refractory period^33^. The reason for the lack of spike generation is the deactivation of ion channels. To address this issue, a highly compact electronic circuit has been developed that can implement the leaky integrate-and-fire model of artificial neurons (Fig. 3c). The leaky and integrated characteristics of the model are implemented through the use of an RC pair. The capacitor (C) integrates the incoming current spikes, while the resistor (R = R_1_ + R_2_) allows the charge to leak out during the time intervals between spikes. The crucial firing feature of the model is achieved by setting the voltage threshold of the SCR through its anode-cathode tension, which is adjusted by the gate via resistors R_1_ and R_2_. Once the voltage threshold is reached, the SCR switches to the on-state, and the capacitor discharges rapidly through the small R_3_, producing a spike of current. The SCR remains in the on-state until the current decreases to the value of I_hold_, which occurs when the capacitor is nearly fully discharged^21^. The observed process can be correlated with the relaxation or refractory period of the artificial neuron. To ensure that the spike can activate a downstream neuron, the strength of the signal must be enhanced. Therefore, we propose a design for an ultracompact (UC) neuron that uses only one SCR and two transistors, in addition to a "membrane" capacitor and several resistors. This construction has a minimal number of components. Specifically, we assigned each of the three features of the LIF model to three respective devices: a resistor, a capacitor, and an SCR. These components enable the nonlinear process of threshold spike generation in the "soma" of the artificial neuron. The change in membrane potential under a limiting input pulse is given in Fig. S8. Figure 3d illustrates the simulation outcomes of the very large scale integration (VLSI) circuit when subjected to different types of excitatory inputs, namely, a train of synaptic transistor pulses^34^. Consistent with LIF neuron behavior, increasing the pulse amplitude results in a decrease in the number of pulses required to trigger a response. The three curves (red, green, and blue) demonstrate that the circuit necessitates 10 pulses of 1 μA and 8 pulses of 1.25 μA to reach the threshold.

### Adaptation between dataset and SNN

**Fig. 4:**
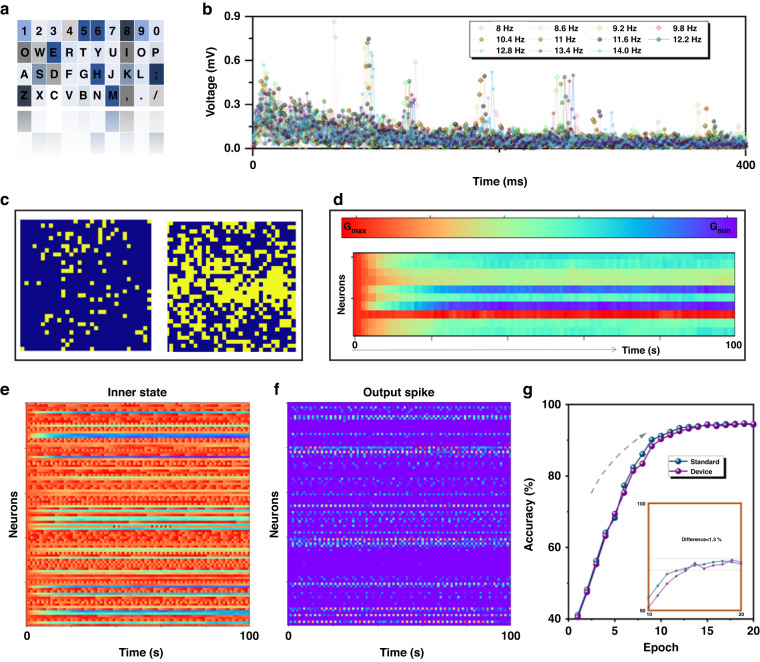
The SSVEP identification task is based on the modified SNN. **a** Steady-state visual evoked potentials (SSVEPs) are a favored signal in brain-computer interface (BCI) systems due to their high information transfer rate (ITR). **b** Eight types of electroencephalographic (EEG) waveforms from 40 different frequencies in the same channel. **c** SSVEP is processed into a visualized two-dimensional matrix through temporary coding. **d** Synaptic efficacy (conductance of channel), defined as the strength of the communication between neurons, undergoes temporal modulation characterized by both facilitatory and inhibitory changes. **e** Inner state of neurons based on STDP in SNNs. **f** Output spike of neurons in SNNs. **g** Test accuracy of the recognition task for SSVEPs in the improved network

SSVEP (steady-state visual evoked potential) is a type of brain activity that occurs in response to repetitive visual stimuli with a fixed frequency (Fig. 4a)^33^. One main advantage of SSVEP-based brain-computer interfaces (BCIs) is their high information transfer rate (ITR) (Fig. S9), which refers to the amount of information that can be transmitted per unit of time. In terms of data characteristics, SSVEP signals are typically characterized by a strong response at the stimulus frequency and its harmonics, which can be easily detected and separated from background EEG activity. SSVEP signals are also highly reproducible across trials and participants, which allows for reliable classification and decoding^34^. Furthermore, SNNs are biologically inspired models that mimic the behavior of neurons in the brain and have been shown to be particularly well suited for processing spatiotemporal data such as EEG signals. Figure 4b directly demonstrates the 11 EEG curves within 400 ms under different stroboscopic stimuli (8 Hz–14.0 Hz). Further analysis of different samples reveals that SSVEPs possess the following advantages for neural computation: wide frequency selectivity range, high amplitude response, sensitivity to stimulus brightness and contrast factors, high stability across different experimental repetitions, and modulation by various cognitive tasks. These above characteristics make SSVEPs a valuable tool for applications in human-computer interaction, brain-machine interfaces, and biofeedback^35^. Temporal coding is a neural coding scheme that encodes information through the precise timing and pattern of action potentials, or spikes, in individual neurons or groups of neurons (Fig. 4c). SSVEPs are processed into a visualized two-dimensional matrix through temporary coding. The temporal coding for SSVEPs with different gains (0.25 and 1) and the distribution of neurons in the input layer combined with synaptic characteristics are analyzed in Figs. S10 and S11. Temporal coding plays a significant role in neural information processing and is thought to be particularly important for encoding information such as sound, visual stimuli, and motor commands^36^. One adopted method is rate coding, where the frequency of spikes within a given time window is used to convey information about the intensity or duration of a stimulus. Another improved method is phase coding, where the timing of spikes relative to a particular phase of a periodic stimulus is used to encode information^37,38^. We conducted an experimental demonstration of hardware-in-the-loop training using a prototype synaptic device-based simulation environment. This environment includes a tested array of ZIF-67 synaptic transistors with a conductance response. The purpose of our experiment was to verify the efficacy of our proposed training approach. The results of our study provide evidence of the successful implementation of our hardware-in-the-loop training method, which can be a promising approach for developing more efficient and effective neuromorphic computing systems. The full input layer with different SSVEP frequencies is shown in Fig. S12. The conductances of the synaptic devices are iteratively adjusted during training by the framework through STDP update rules, with communication established between the LIF neurons (Fig. 4d). The synaptic weights of the SNNs are implemented using a double differential configuration with two devices, where G^+^ and G^−^ represent the synaptic weight in proportion to the difference in their conductances (w = G^+^−G^−^). To increase the weight w, the conductance of G^+^ is increased, while to decrease the weight w, the conductance of G^−^ is increased. The conductance of the device increases gradually through ion migration in the ZrO_x_ layer by applying low-power pulses, which allows for gradual weight updates (∆w) during training. The preprocessed sample is subsequently transmitted to the network, causing the input neurons to spike, as illustrated in Fig. 4e. The resulting spike rasters for the output layer neurons during the speech recognition procedure are displayed in Fig. 4f. The inner state value of neurons for layer 1 with different LTP/LTD curves and the inner state value of neurons for layer 0 with different STDPs are both demonstrated in Figs. S13 and Figs. S14. To evaluate the feasibility of the proposed network, the difference between the recognition rate of the standard network and the enhanced network is compared (Fig. 4g). The best recognition rate is still our proposed structure according to the relationship between accuracy and number of layers (Fig. S15). The ultimate arrangements of synapses proved to be effective and showed that the method can be utilized for creating an analog core that serves as a very efficient in-memory inference engine without relying on the von Neumann architecture. Furthermore, to verify the low energy consumption of the synaptic device, the energy consumption per spike of the synaptic transistor is calculated by the Equation E = I_peak_ × t × V = 4.05 pJ (Fig. S16). I_peak_ is the maximum value (2.65 nA) of the generated EPSC curve, t is the spike duration (30 ms), and V is the voltage applied to the drain electrode (0.05 V).

## Conclusion

The novel concepts put forward have opened up numerous opportunities for combining synaptic devices with neuron circuits to serve as the core components of SNNs. This improved SNN, based on the LIF model, offers advanced biologically inspired neural models with low computational complexity and simplicity, enabling exploration of their capabilities from a deep learning perspective. Furthermore, the ZIF-67 SNN system provides a new framework for modeling and understanding neural dynamics, which can benefit from memory, synaptic plasticity, and membrane potential, from a neuroscientific perspective. The in-memory accelerators, combined with the SNN-based STDP weight update rule, offer the potential for high adoption rates of spiking neural networks for SSVEP recognition applications (with a rate of 95.1%) and enable power-efficient neuromorphic hardware implementations from a neuromorphic computing perspective. Finally, the integration of multifunctional synaptic transistors into the improved SNNs expands the use of existing or forthcoming network accelerators for the entire SNN implementation and deployment.

## Methods

### Synthesis of transistors

The solution of ZrO_x_ precursor was prepared by dissolving 1.5 M aluminum nitrate hydrate (Zr(NO_3_)_2_·xH2O) in 20 mL of 2-methoxy ethanol (2-Me). To obtain the ZrO_x_-Li precursor solution, 1.5 M aluminum nitrate hydrate (Zr(NO_3_)_2_·xH2O) and 0.15 M indium nitrate hydrate were mixed in 20 mL of deionized water. The InO_x_ precursor solution was prepared by dissolving indium nitrate hydrate (In(NO_3_)_3_·xH2O) in 20 mL of deionized water. All solutions were vigorously stirred for 5 h under atmospheric conditions and then filtered using 0.25 μm PTFE syringe filters before spin coating. In a typical process, 1.0 mmol of cobalt nitrate hexahydrate and 4.0 mmol of 2-methylimidazole were dissolved in 15.0 mL of methanol. The 2-methylimidazole solution was then slowly poured into the cobalt nitrate hexahydrate solution under stirring for 6 h. The mixture was aged at room temperature for 16 h, and the purple precipitate obtained was collected by centrifugation, washed with methanol, and dried at 70 °C for 12 h. The ZIF-76 (purple powder) obtained was characterized by XRD^3^. Finally, the obtained mixture was centrifuged at 3500 rpm for 3 min to obtain a black‒brown few-layer dispersion of approximately 5 mg/mL, which was stored in an argon atmosphere for up to 14 days.

### Fabrication of synaptic transistors

First, a heavily doped Si (n^++^) substrate was cleaned by deionized water and dried under N_2_ flow. Afterward, the processed substrate was further treated by plasma for 15 min to allow the film surface hydrophilic treatment. The ZrO_x_ and ZrO_x_-Li films were spin-cast with precursor solution at 4500 rpm for 30 s and then annealed for 80 min at 250 °C in an air atmosphere. Then, the ZIF-67 solution was diluted to 1 mg/mL and spin-coated at 3000 rpm for 20 s on the surfaces of ZrO_x_ and ZrO_x_-Li films.^6^ Substrates with solution film were then oxidized at 80 °C for 1 min on a hotplate in air. The InO_x_ film was spin-cast with precursor solution at 3500 rpm for 30 s and then annealed for 1 h at 200 °C in an air atmosphere. The 30 nm thick Al source/drain (S/D) electrodes were fabricated by thermal evaporation through the shadow mask^38^.

First, a heavily doped silicon (n^++^) substrate underwent a cleaning process using deionized water and was subsequently dried under a flow of nitrogen gas. The substrate then received an additional plasma treatment lasting for 15 min, which allowed for hydrophilic treatment of the film surface. Next, ZrO_x_ and ZrO_x_-Li films were applied to the substrate via spin-casting a precursor solution at 4500 rpm for 30 s, followed by an 80-minute annealing process at 250 °C in an air atmosphere. Next, a ZIF-67 solution was diluted to a concentration of 1 mg/mL and spin-coated at 3000 rpm for 20 s onto the surfaces of the ZrO_x_ and ZrO_x_-Li films. The substrate with the solution film was then oxidized at 80 °C for 1 min on a hotplate in an air atmosphere. Additionally, an InO_x_ film was applied to the substrate via spin-casting a precursor solution at 3500 rpm for 30 s, which was subsequently annealed for 1 h at 200 °C in an air atmosphere. Finally, 30 nm thick aluminum source/drain (S/D) electrodes were created through thermal evaporation using a shadow mask^39^.

### Characterization of synaptic plasticity

The electrical properties of the Al/InO_x_/MXenes/ZrO_x_-Li/Si/Al synaptic transistor were tested using a semiconductor parameter analyzer with transistor characterization software under atmospheric conditions. To measure the EPSC and LTD/LTP current flowing between the source/drain (S/D) electrodes, a 0.1 V steady voltage bias was applied to the postsynaptic terminal (V_post_). The chemical compositions of the dielectric and semiconductor layers were determined using X-ray photoelectric spectroscopy (XPS)^4^. The crystallization and structural information of the thin films were obtained using X-ray diffraction (XRD) with Cu Kα radiation (λ = 1.542 Å).

### SNN simulation

Before implementing the SNN, we need to transform signals in the time domain into time encoding forms with different time intervals. One commonly used time encoding methodology is time-to-first spike coding. This encoding method represents information as the release time of the first pulse of a neuron. Assuming that the neuron only generates one pulse, the neuron is in a suppressed state until the next stimulus arrives. The time of generating the pulse is proportional to the value of the analog quantity, indicating that the time of the first pulse generated after receiving the stimulus contains all the information of the stimulus.

We use SNNs as the base model, which is a compact and efficient neural network. After we converted the SSVEP data into two-dimensional matrix data, we could directly pass it into the block. The LIF block is the main component of SNNs. Compared with the ordinary neural network structure, the LIF and STDP block can not only perform weighted operations through the convolution layer and activation function mechanism to extract features but also retain the initial information of the input data and fuse it with the obtained feature information^11^. Two blocks and one linear layer are used in our model. The input image data are passed through two residual blocks to complete the feature extraction and then passed to the linear layer to complete the final classification task. Usually, this is a complete SNN workflow, and we use a standard learning rate in the training step. After each training of the network, different learning steps are used to update the network parameters according to the change direction of the loss. Our simulation process is based on the SSVEP Database. An SSVEP database is a collection of SSVEP signals recorded from different subjects under different experimental conditions. This SSVEP database contains a database of 8-channel EEG data from 30 subjects performing a 4-target SSVEP-BCI task.

In the cross-bar array of synaptic transistors, the calculated conductance was used to apply a positive synaptic weight value. However, in the measurement of neurocomputing in SNNs, negative values are also included. Therefore, the synaptic weight (W = G^+^−G^−^) was defined as the difference between the states of two synaptic devices (represented by G^+^ and G^−^) for each conductance value. The initial weights were set to random fluctuations near 0, and the values between G_min_ and G_max_ were normalized to (−1, 1). The actual updated weight value depended on the difference between the conductance states of the two synaptic devices (G^+^ and G^−^) extracted from the LTP/LTD curve. The synaptic weight was defined as the difference in conductance between two synaptic transistors representing a single neuron. If sgn(ΔW) > 0, then the Formula W ↑ = G^+^ ↑-G^−^ ↓ was used, while if sgn(ΔW) < 0, then the Formula W ↓ = G^+^ ↓-G^−^ ↑ was used. The reference circuit is based on the TSMC 65 nm CMOS process^40^.

## Supplementary information

Supporting Information
