# Supplementary material for: Synaptic transistor with multiple biological functions based on metal-organic frameworks combined with the LIF model of a spiking neural network to recognize temporal information
Source: Microsyst Nanoeng. 2023 Jul 21;9:96. doi: 10.1038/s41378-023-00566-4 (PMC10362020; doi:10.1038/s41378-023-00566-4)
Supplement: Supplementary file 1 — Supporting Information [file 41378_2023_566_MOESM1_ESM.docx]

**Supporting Information**

**Synaptic Transistor with Multiple Biological Function Based on Metal-Organic Frameworks Combined with LIF Model of Spiking Neural Network to Recognize Temporal Information**

*Qinan Wang^1,3^, Yi Sun^1,3^, Rongxuan Xu^1,3^, Chenran Li^1,3^, Chengbo Wang^1,3^, Wen Liu^1,^*,Jiangmin Gu^1^, Li Yang^4^, Xin Tu^4^, Hao Gao^5^, Chun Zhao^1,^*, Zhen Wen ^2,^**

^1^School of Advanced Technology, Xi'an Jiaotong-Liverpool University, Suzhou 215123, China

^2^Institute of Functional Nano and Soft Materials (FUNSOM), Joint International Research Laboratory of Carbon-Based Functional Materials and Devices, Soochow University, Suzhou 215123, P.R. China.

^3^Department of Electrical Engineering and Electronics, University of Liverpool, Liverpool L69 3GJ, UK

^4^School of Science, Xi'an Jiaotong-Liverpool University, Suzhou 215123, China

^5^Department of Electrical Engineering, Eindhoven University of Technology, Den Dolech 2, 5612 AZ, The Netherlands

^*^Correspondence E-mail: [Chun.Zhao@xjtlu.edu.cn](mailto:Chun.Zhao@xjtlu.edu.cn) (C. Zhao); [Wen.Liu@xjtlu.edu.cn](mailto:Wen.Liu@xjtlu.edu.cn) (W. Liu); [wenzhen2011@suda.edu.cn](mailto:wenzhen2011@suda.edu.cn) (Z. Wen)

**
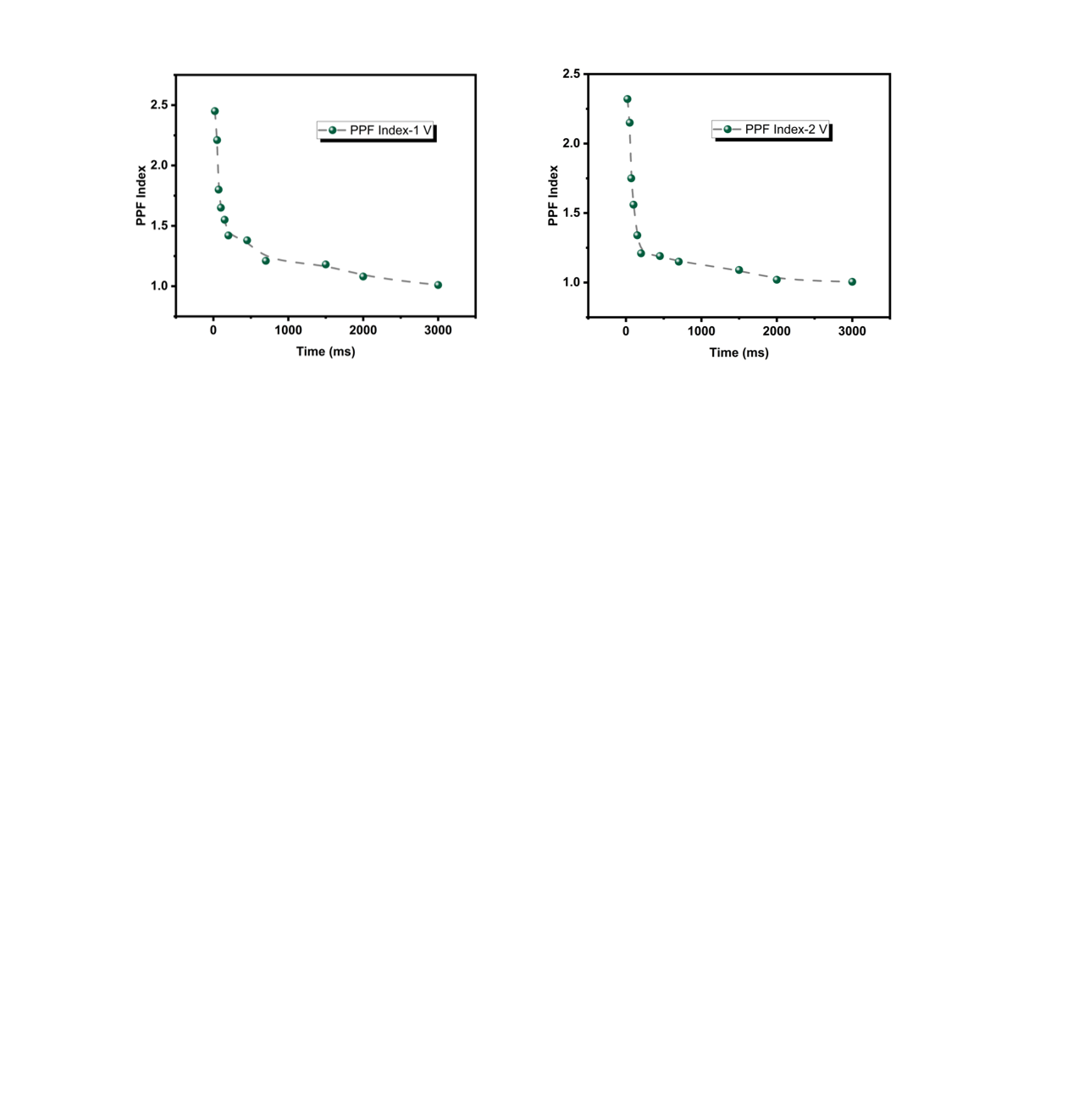
Fig. S1**. PPF curves under different voltage (1 V and 2 V) conditions.

**
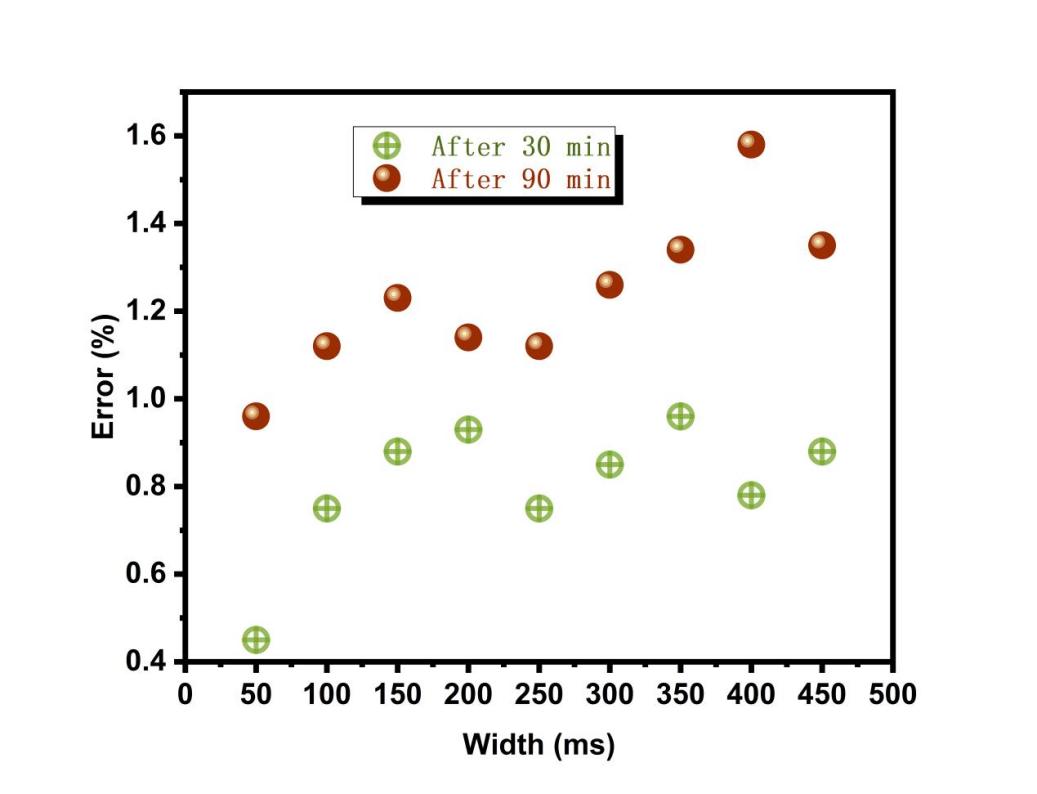
**

**Fig. S2**. The long-term retention of synaptic plasticity after 30 minutes and 60 minutes.

**
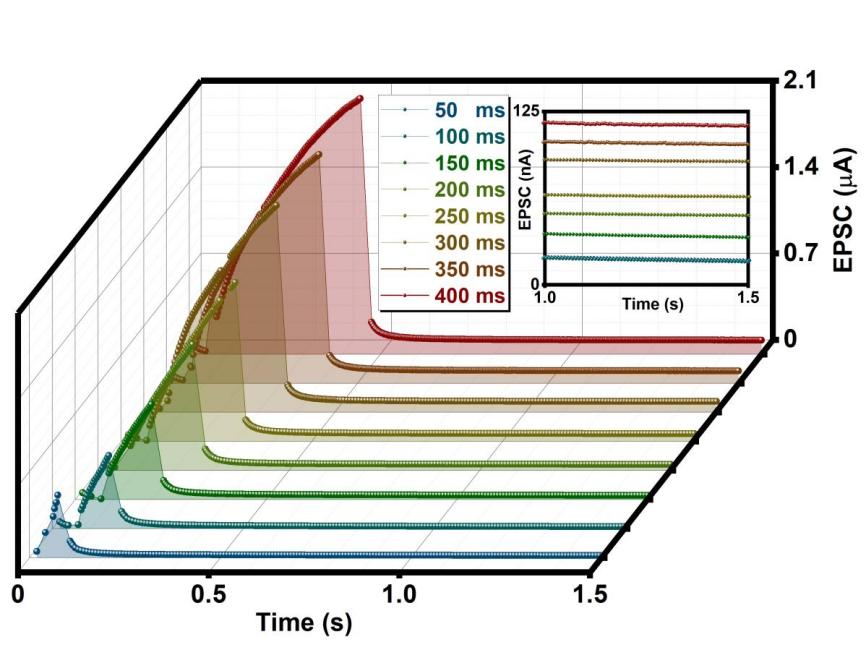
**

**Fig. S3**. Typical EPSC when Li ion doping concentration is 5 %.

**
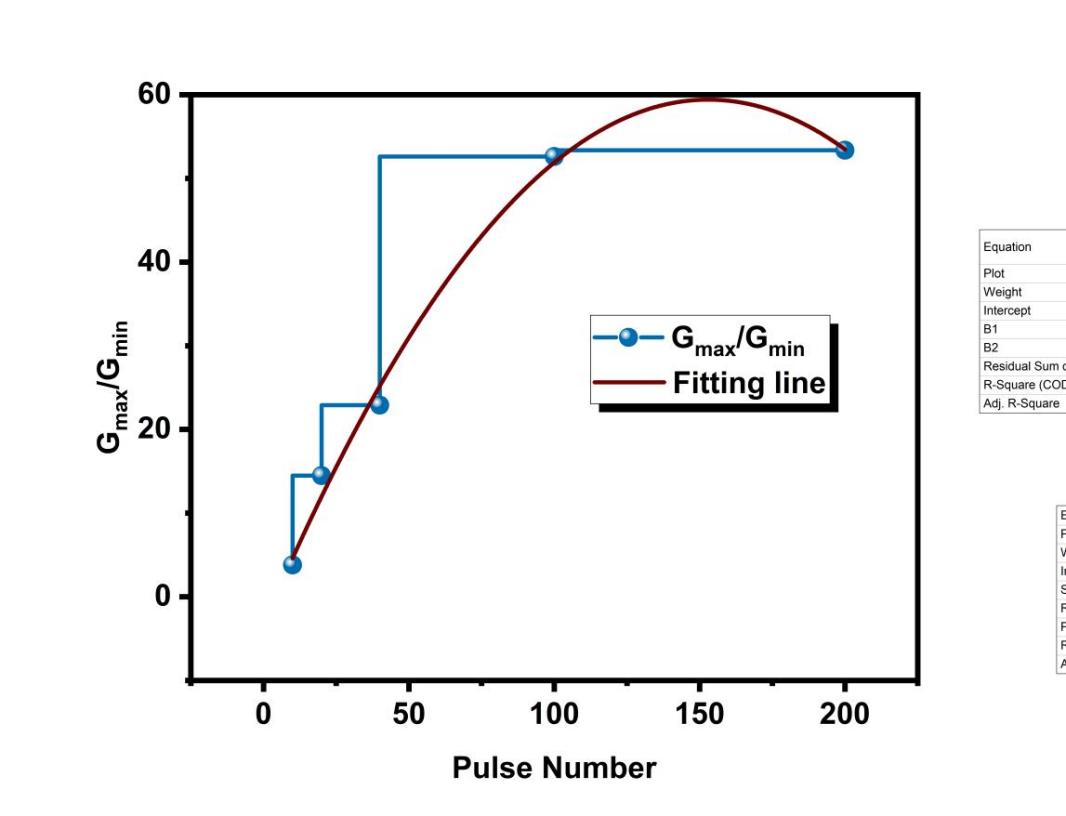
**

**Fig. S4**. The G_max_/G_min_ ratio of 10, 20, 40 100, and 200 LTP/LTD curves.

**
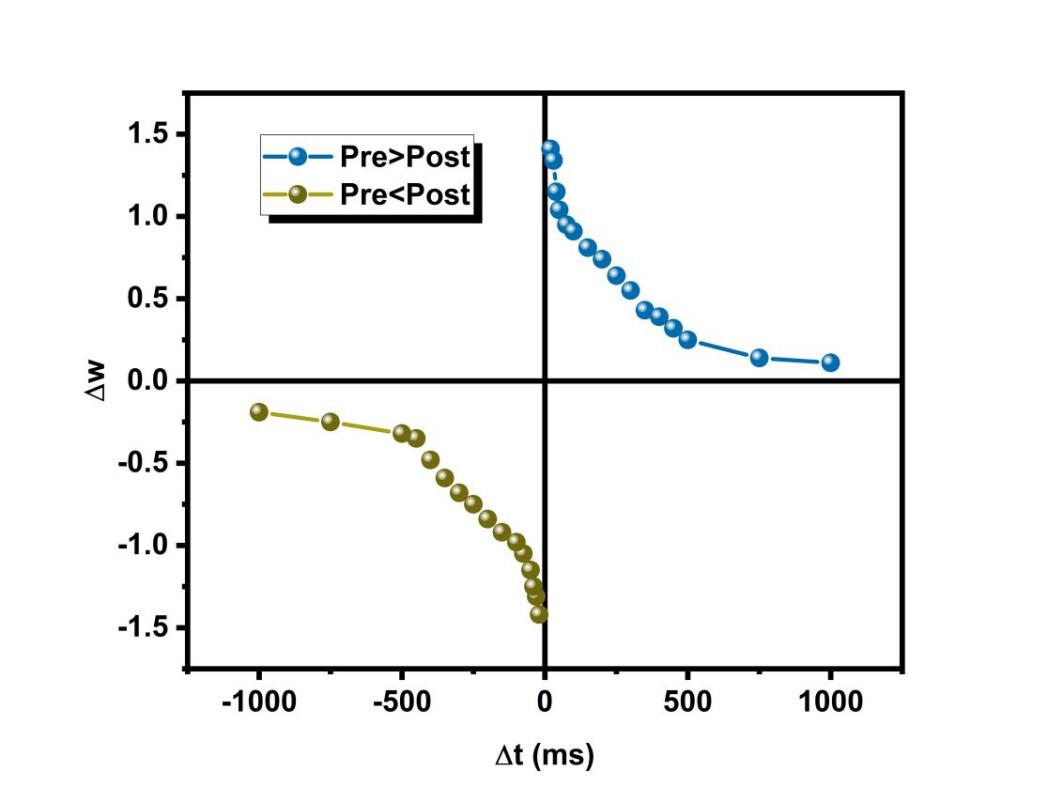
**

**Fig. S5**. The minimum voltage that can trigger the STDP

**
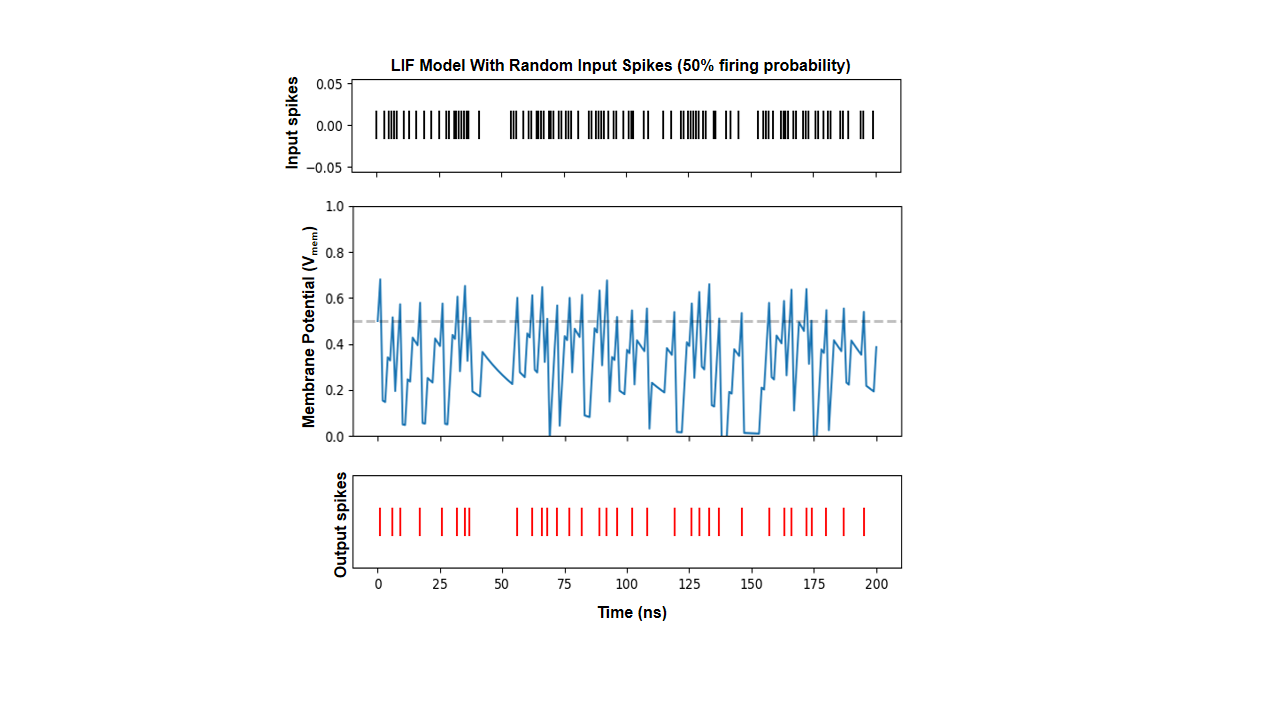
**

**Fig. S6**. LIF model with random input spikes (50% firing probability).

**
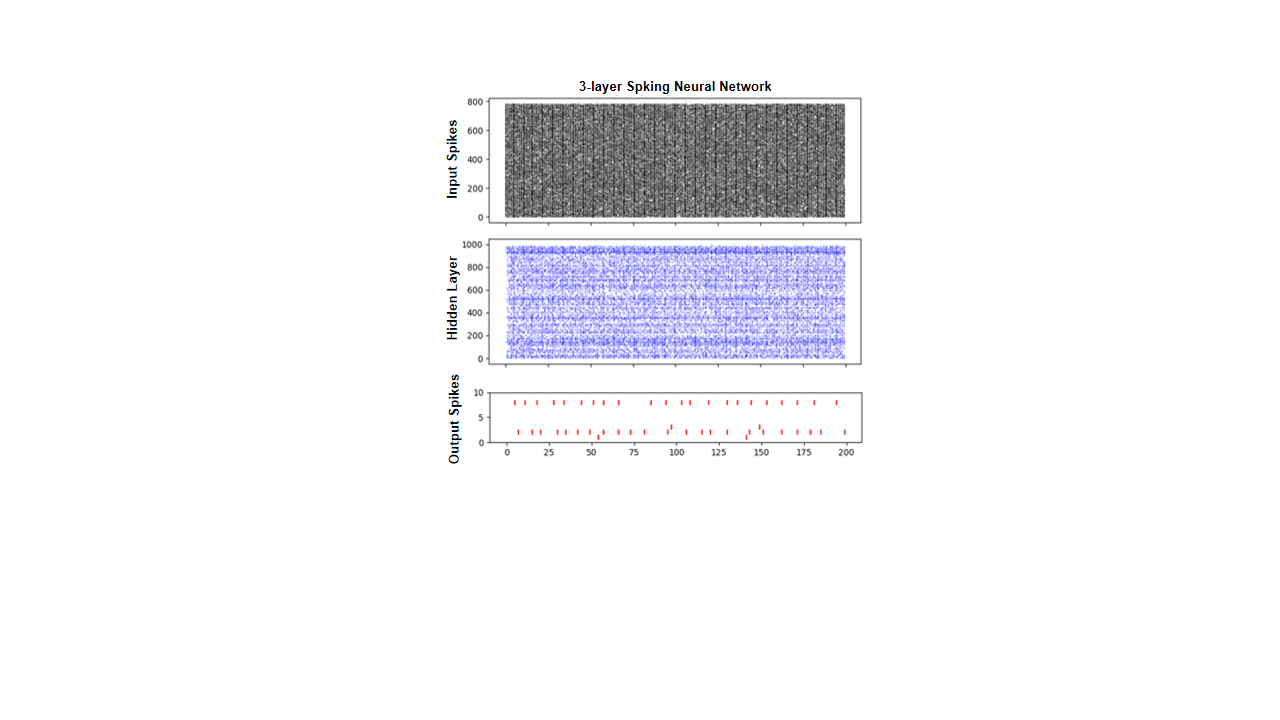
**

**Fig. S7**. 3-layer of improved Spiking Neural network based on the STDP of synaptic transistor.


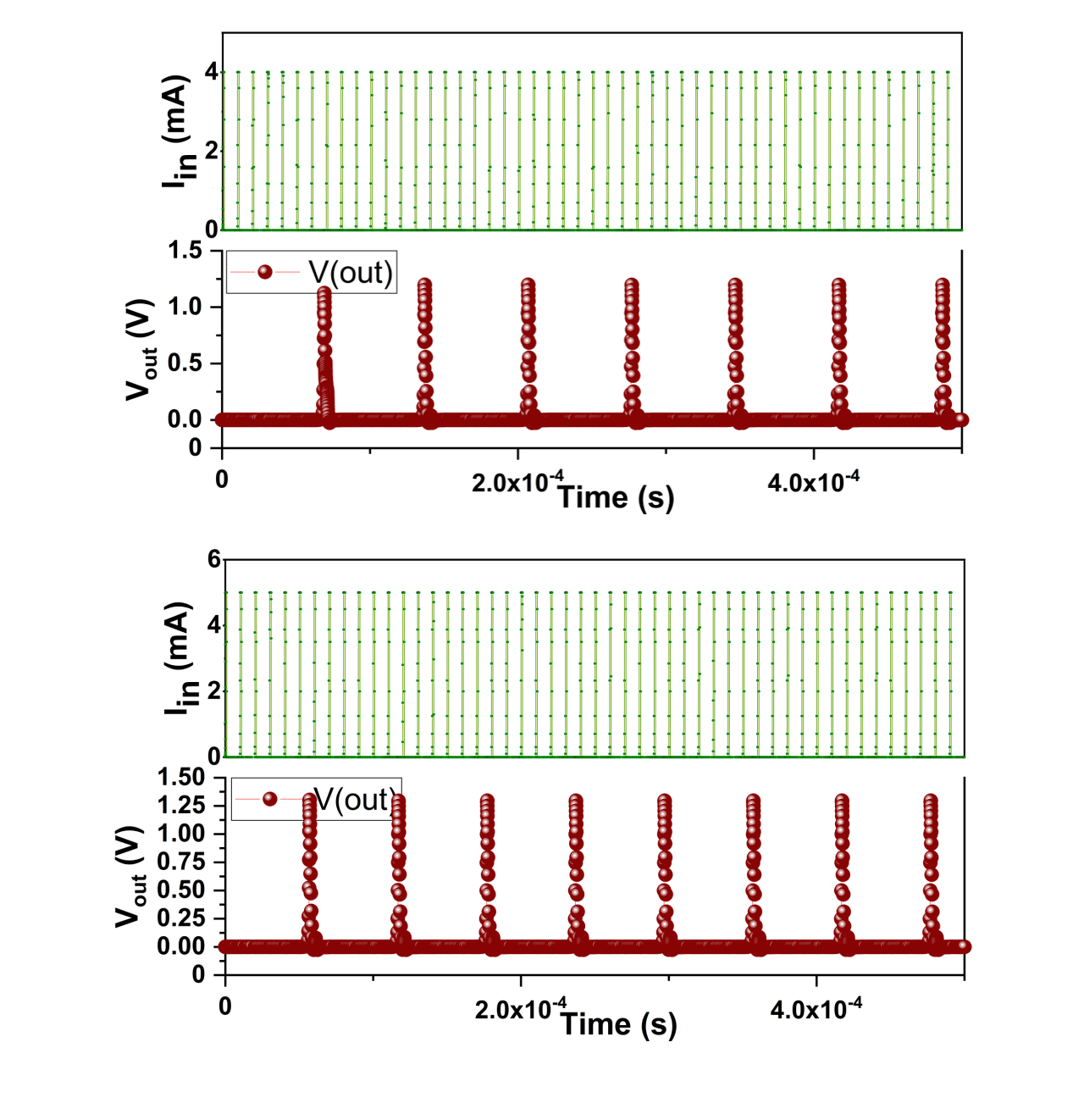
**Fig. S8**. Change of membrane potential under different input pulses.

**
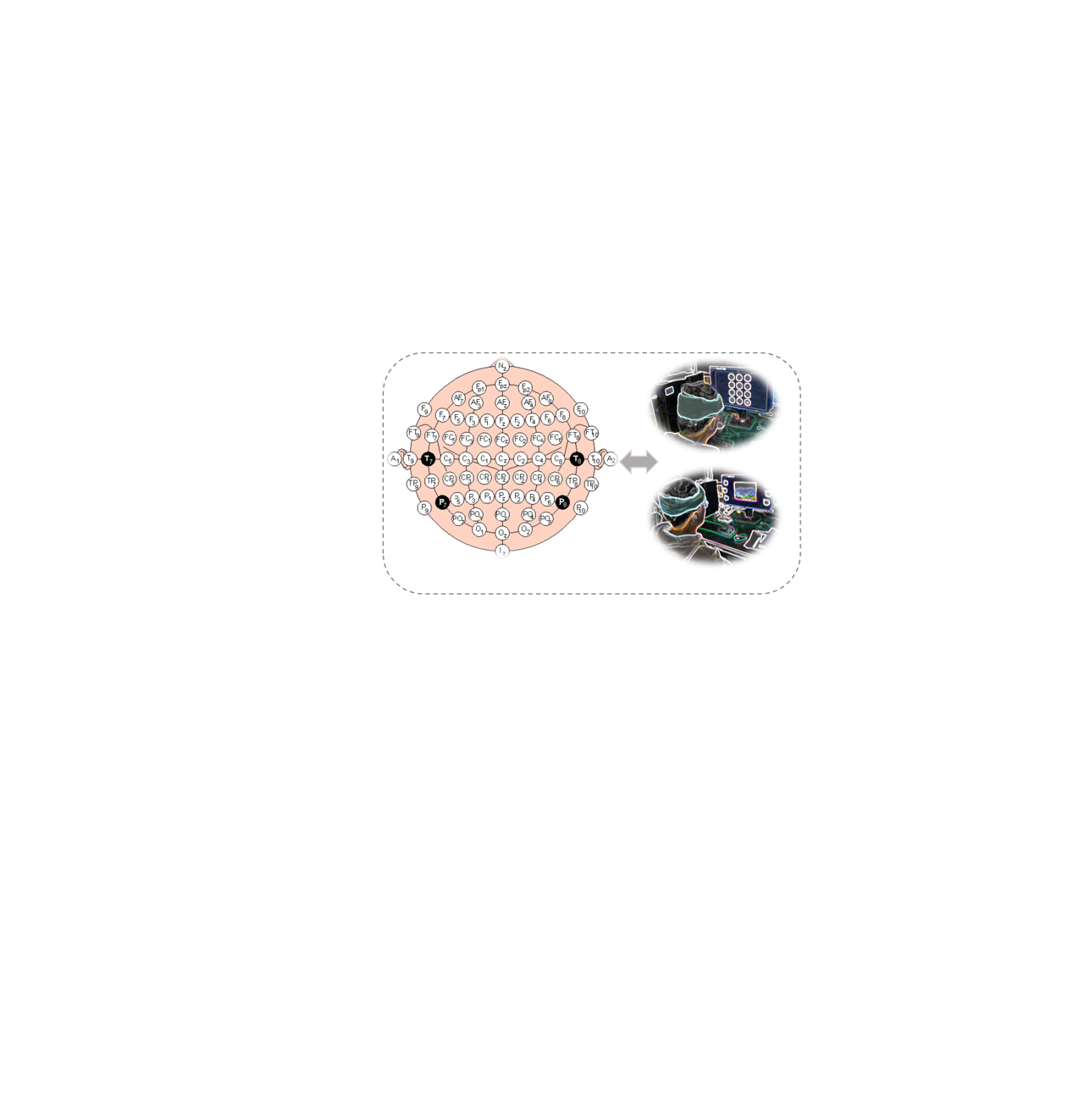
**

**Fig. S9**. Process of extracting SSVEP dataset.

**
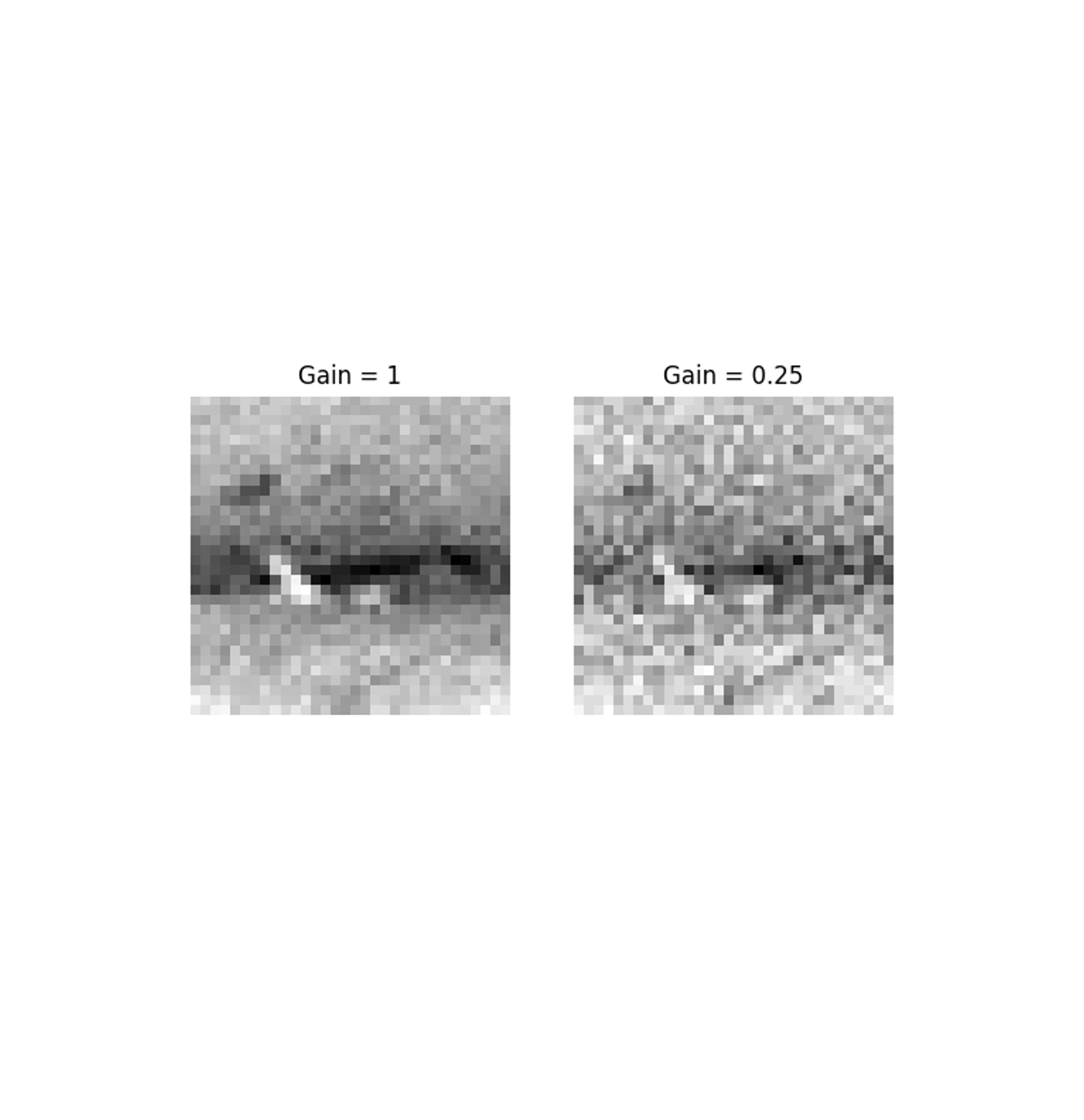
**

**Fig. S10**. The Temporal coding for SSVEP with different gain (0.25 and 1).

**
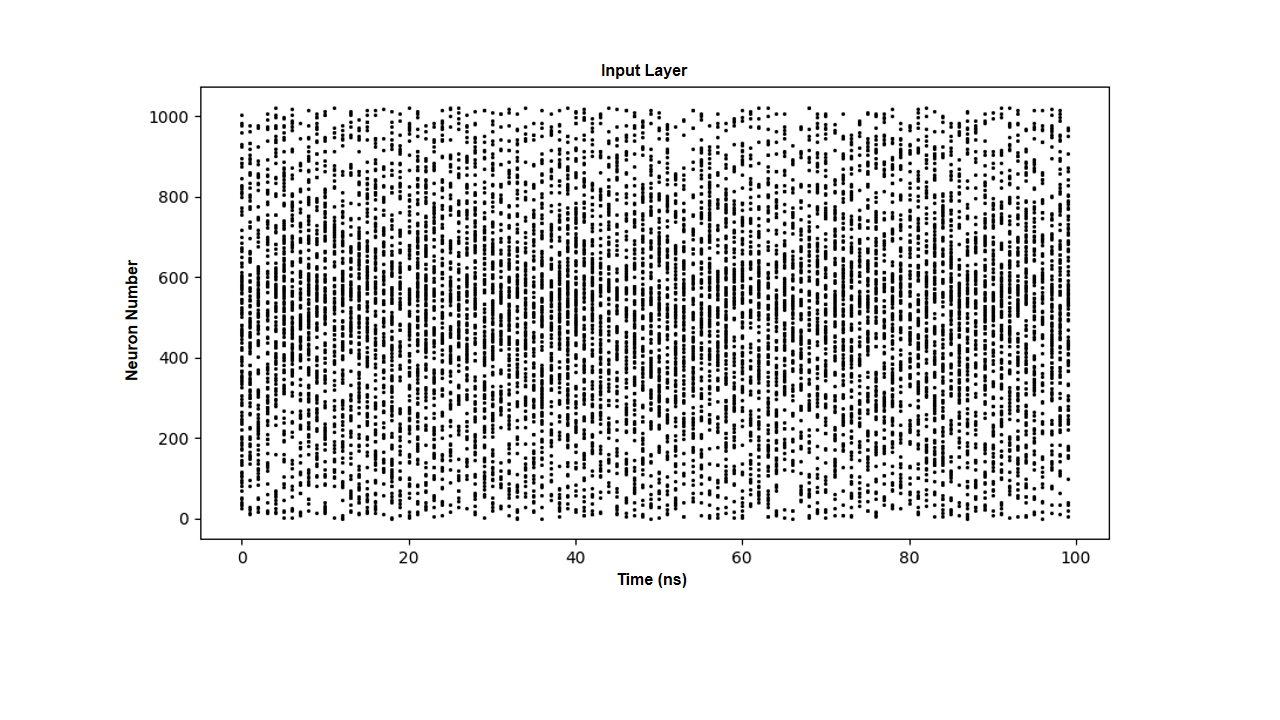
**

**Fig. S11**. The distribution of neurons in the input layer combined with synaptic characteristics.

**
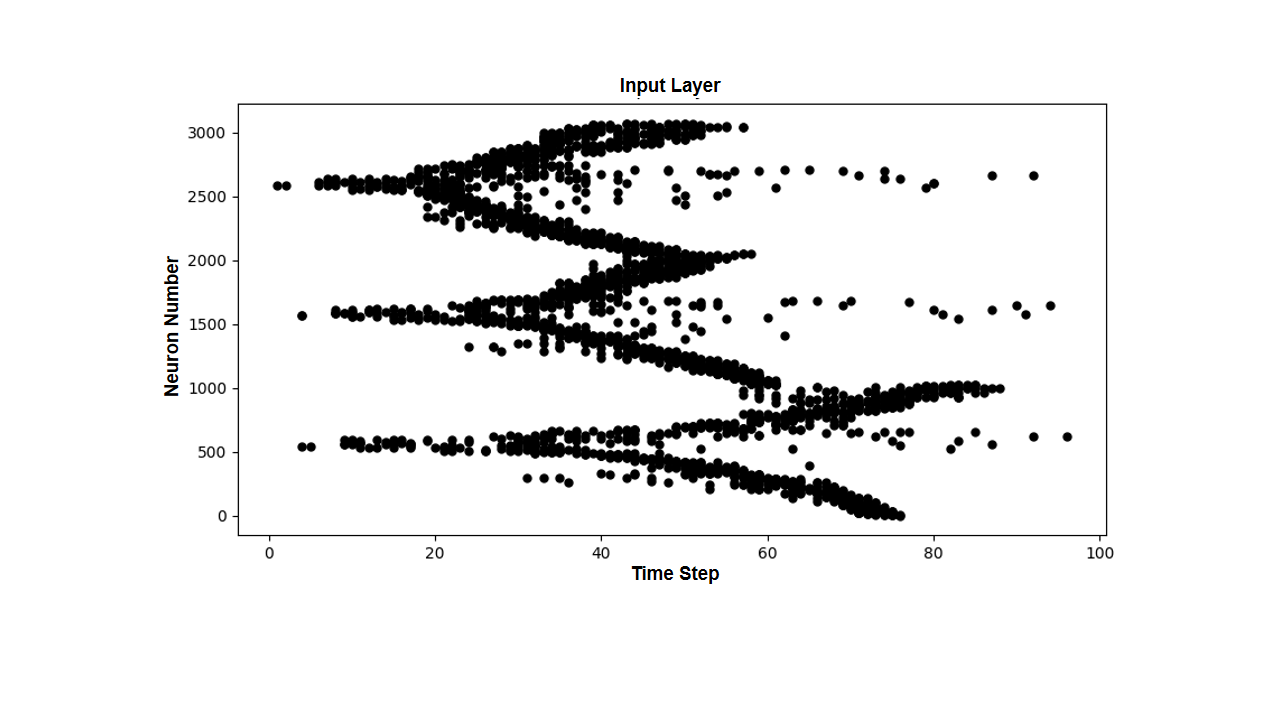
**

**Fig. S12**. The full input layer with different SSVEP frequency.


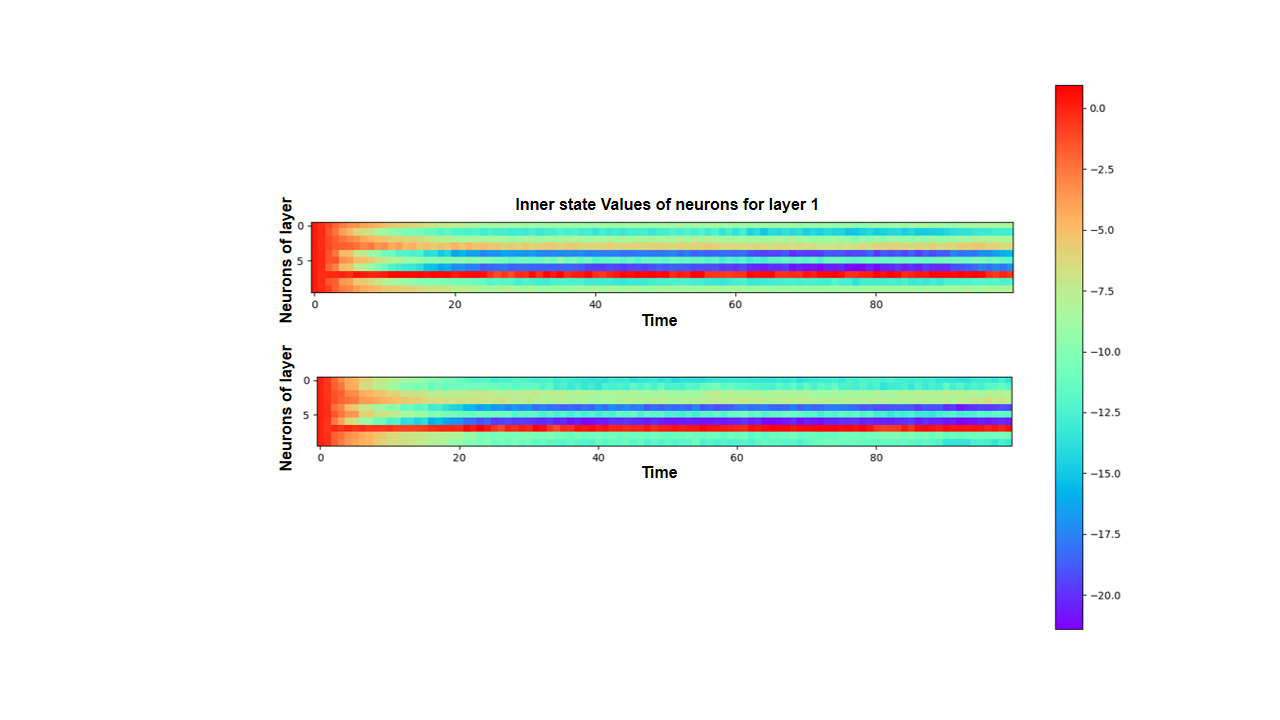


**Fig. S13**. Inner state value of neurons for layer 1 with different LTP/LTD.

**
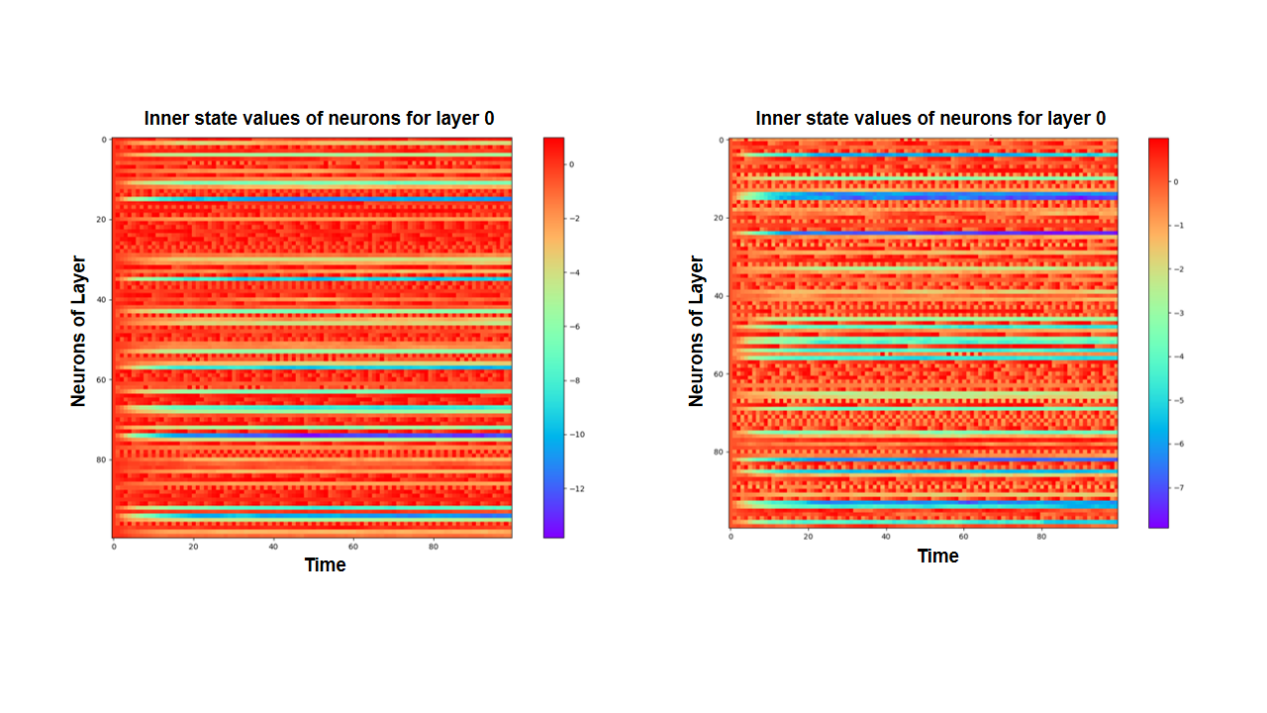
Fig. S14**. Inner state value of neurons for layer 0 with different STDP.

**
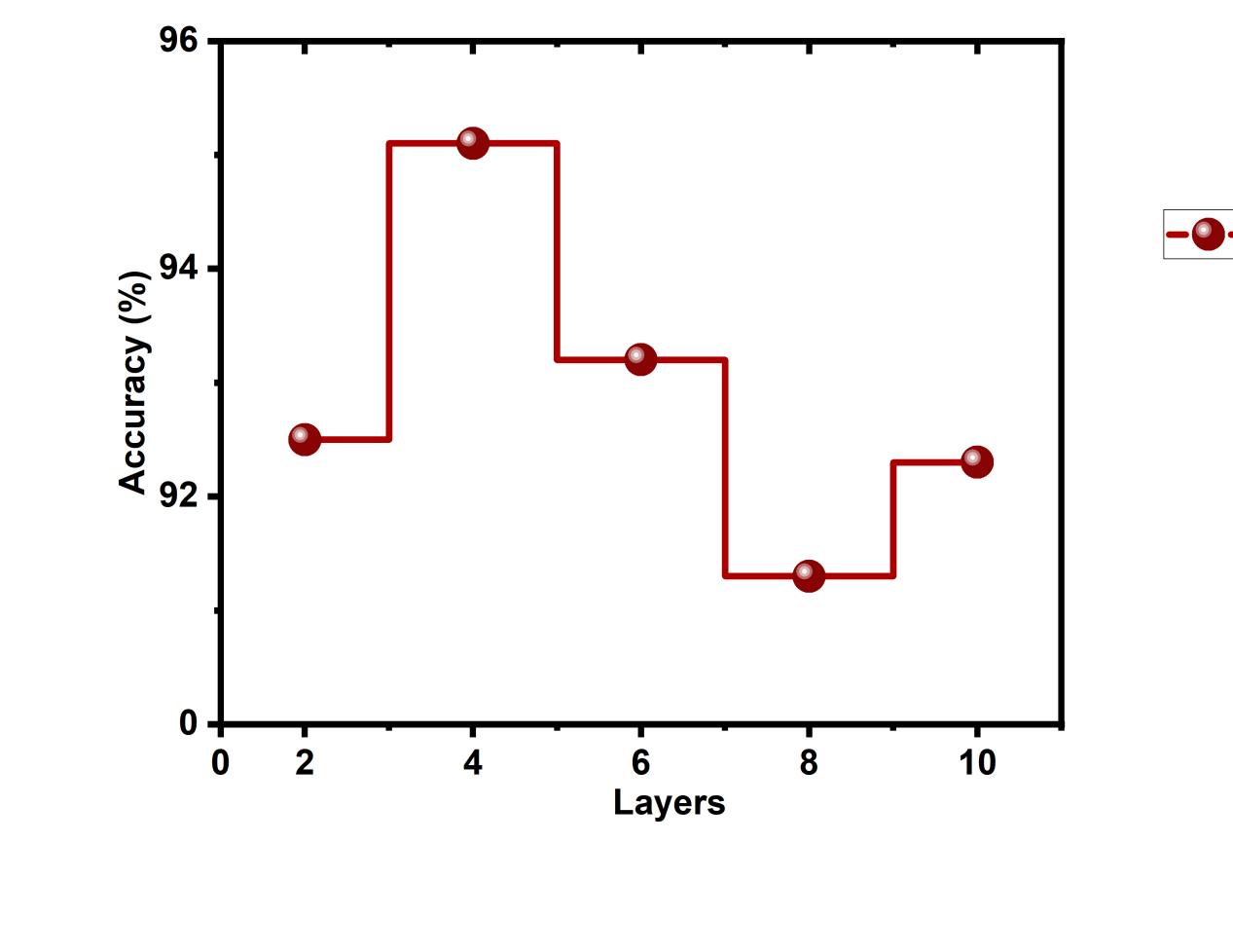
Fig. S15**. The influence of different layers on recognition rate in SNN.


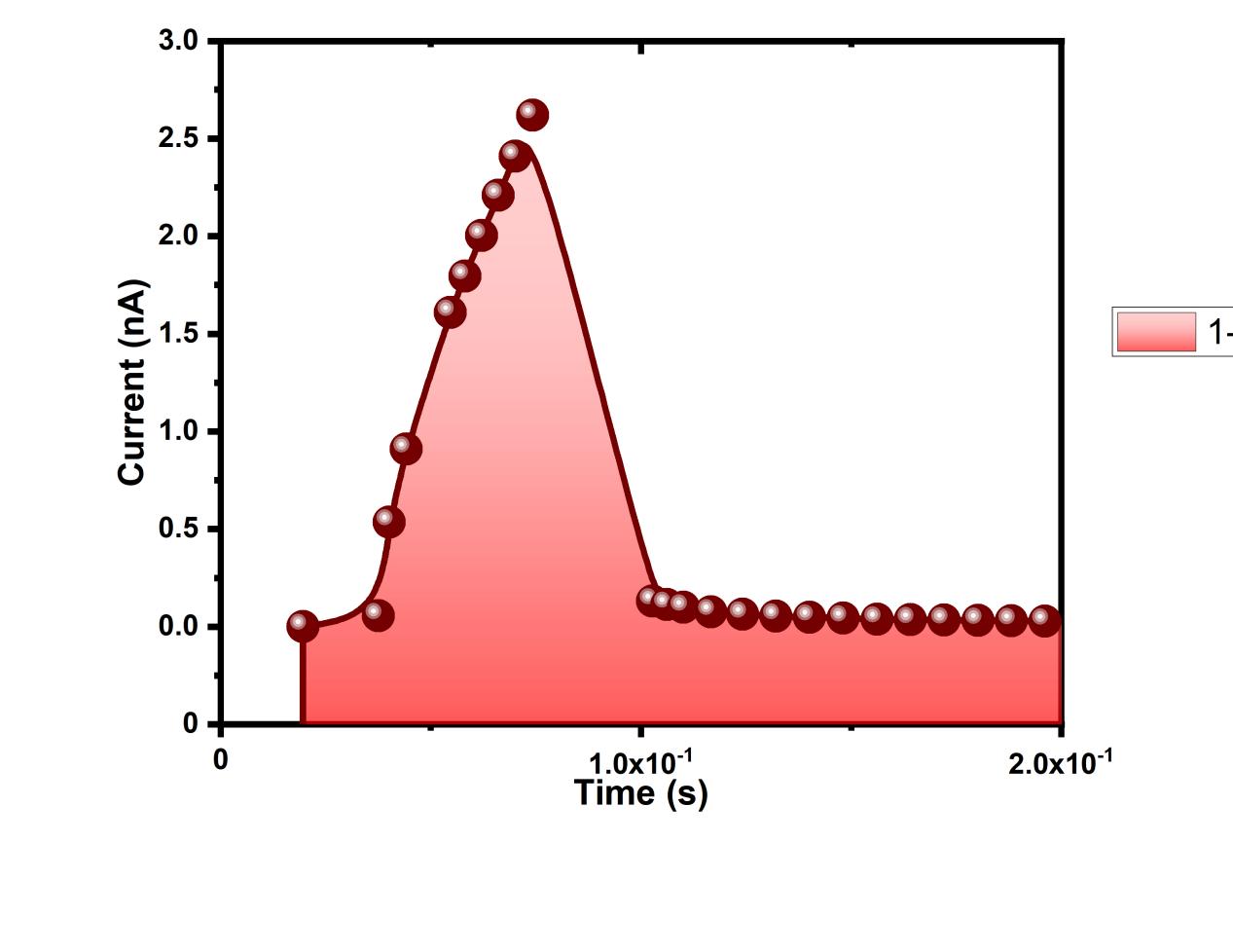
**Fig. S16**. EPSC property triggered by presynaptic spike (V_gs_=0.5 V, V_ds_=0.05 V, Pulse Width=30 ms) in 0.2 s.
